# RETRACTION: Revealing an Association Between HPV and Systemic Lupus Erythematosus: A Bidirectional Two‐Sample Mendelian Randomization Study

**DOI:** 10.1111/srt.70178

**Published:** 2025-05-16

RETRACTION: F. Pan, H. Shen, B. Wang, and J. Wang, “Revealing an Association Between HPV and Systemic Lupus Erythematosus: A Bidirectional Two‐Sample Mendelian Randomization Study,” Skin Research and Technology 30, no. 8 (2024): e13913, https://doi.org/10.1111/srt.13913.

The above article, published online on 7 August 2024 in Wiley Online Library (wileyonlinelibrary.com), has been retracted by agreement between *Skin Research and Technology*; and John Wiley & Sons Ltd.

The article was accepted for publication following an insufficient peer review process that does not meet the standards of the journal's peer review policy. Furthermore, during investigation by the editorial team and Wiley's research integrity team, concerns about the study's conceptual framework were identified. Specifically, the article does not adequately present the biological rationale necessary to support the valid application of Mendelian Randomization to the research question. As a result, the editorial team cannot vouch for the validity of the methodological approach employed, nor the reliability of the study's conclusions. Accordingly, the article has been retracted. The authors have been informed of this decision and disagree with it.

